# The Right Coronary Anatomy and Operative Topography of the Tricuspid Valve Annulus

**DOI:** 10.3390/jcdd11060159

**Published:** 2024-05-21

**Authors:** Michał Piotrowski, Marian Burysz, Jakub Batko, Radosław Litwinowicz, Mariusz Kowalewski, Krzysztof Bartuś, Krzysztof Wróbel, Łukasz Graczykowski, Artur Słomka

**Affiliations:** 1 CAROL—Cardiothoracic Anatomy Research Operative Lab, Department of Cardiovascular Surgery and Transplantology, Institute of Cardiology, Jagiellonian University Medical College, 31-008 Krakow, Poland; michal21piotrowski@gmail.com (M.P.);; 2 Department of Cardiac Surgery, Regional Specialist Hospital, 86-300 Grudziądz, Poland; 3 Thoracic Research Centre, Collegium Medicum Nicolaus Copernicus University, Innovative Medical Forum, 85-094 Bydgoszcz, Polandartur.slomka@cm.umk.pl (A.S.); 4 Department of Cardiac Surgery, Central Clinical Hospital of the Ministry of Interior, Centre of Postgraduate Medical Education, 01-813 Warsaw, Poland; 5 Cardio-Thoracic Surgery Department, Heart and Vascular Centre, Maastricht University Medical Centre, Cardiovascular Research Institute Maastricht (CARIM), 6229 HX Maastricht, The Netherlands; 6 Department of Cardiovascular Surgery and Transplantology, Institute of Cardiology, Jagiellonian University Medical College, 31-008 Krakow, Poland; 7 Department of Cardiac Surgery, Warsaw Medicover Hospital, Lazarski University, 02-972 Warsaw, Poland; 8 Department of Cardiology, Specialist Hospital in Wloclawek, 87-800 Wloclawek, Poland; 9 Department of Pathophysiology, Ludwik Rydygier Collegium Medicum in Bydgoszcz, Nicolaus Copernicus University in Toruń, 85-094 Bydgoszcz, Poland

**Keywords:** tricuspid valve anatomy, tricuspid valve repair, tricuspid valve replacement, right coronary artery damage

## Abstract

Background: The region of the tricuspid valve is an important area for various cardiac interventions. In particular, the spatial relationships between the right coronary artery and the annulus of the tricuspid valve should be considered during surgical interventions. The aim of this study was to provide an accurate description of the clinical anatomy and topography of this region. Methods: We analyzed 107 computed tomography scans (44% female, age 62.1 ± 9.4 years) of the tricuspid valve region. The circumference of the free wall of the tricuspid valve annulus was divided into 13 annular points and measurements were taken at each point. The prevalence of danger zones (distance between artery and annulus less than 2 mm) was also investigated. Results: Danger zones were found in 20.56% of the cases studied. The highest prevalence of danger zones and the smallest distances were found at the annular points of the tricuspid valve located at the posterior insertion of the leaflets, without observed sex-specific differences. Conclusion: The highest risk of iatrogenic damage to the right coronary artery is in the posterior part of the tricuspid valve annulus.

## 1. Introduction

### 1.1. Anatomy of the Annulus of the Tricuspid Valve

The normal tricuspid valve is the largest human heart valve that usually has three leaflets (the number can vary from two to seven) [1,2]. It is surrounded by a fibrous, saddle-shaped tricuspid valve annulus (TVA) that separates the right atrium (RA) and the right ventricle (RV) from the myocardium. It can be divided into four main parts: antero- and posterolateral as well as antero- and posteroseptal [1,3]. The geometry of the TVA can be significantly altered by tricuspid regurgitation (TR) [1,4].

### 1.2. Clinical Significance of the Region of the Tricuspid Valve Annulus

The region of the TVA is of clinical importance in various surgical procedures, including tricuspid valve surgery, coronary artery bypass grafting, percutaneous coronary interventions, and ablation procedures [5,6,7,8,9]. Its size, shape, and dynamics may change depending on the pathology of the tricuspid valve and the remodeling of the RA and RV [6,10]. In addition, the septal part of the TVA is adjacent to Koch’s triangle where the atrioventricular node (AVN) is located [1,6,11]. The area adjacent to the TVA and the ostium of the inferior vena cava, the so-called cavotricuspid isthmus, is one of the ablation targets for the treatment of atrial flutter and atrial fibrillation [5,12]. The main structure surrounding the TVA is the right coronary artery (RCA), which vascularizes the RA, the RV, part of the interventricular septum, and, in some cases, part of the left ventricle (LV) [1,3]. Short distances between the RCA and TVA are relatively common in the cavotricuspid isthmus (in addition to proximity to the RCA) [13]. According to a study by Al Aloul et al., the distance is less than 5 mm in 80% of patients, which may have an impact on the vulnerability of this area to damage during tricuspid annuloplasty [13]. The TVA region, referred to in the nomenclature as the right atrioventricular coronary sulcus, can be accessed via various endocardial venous approaches or via a direct epicardial approach [7,8,9].

### 1.3. Indications and Complications of Tricuspid Valve Surgery

Tricuspid annuloplasty is a standard surgical treatment for TR. Tricuspid valve annuloplasty can be performed either percutaneously, minimally invasively, or via a sternotomy approach [7,8,9,10,14]. Unfortunately, the newer percutaneous devices require sufficient space around TVA as well as tissue with optimal durability for their implantation [9,15]. The potential complications of minimally invasive access, such as tissue dehiscence and RCA damage, may be related to the anatomy of this region where the TVA and RCA are close to each other, as well as fragile tissue [13,15]. Damage to the RCA is rare but a serious complication in both approaches to tricuspid annuloplasty is possible [15]. To potentially reduce the likelihood of the previously described problems, a thorough topographic examination of the TVA zone should be performed.

### 1.4. The Aim of the Study

The aim of the study was to describe the morphometry and topography of the tricuspid annulus region with regard to the interrelationship with the RCA in the context of tricuspid annuloplasty and to determine the surgical danger zones.

## 2. Materials and Methods

### 2.1. Study Population

Contrast-enhanced, electrocardiogram-guided computed tomography angiography (CTA) scans of 107 patients with atrial fibrillation (44% female, age 62.1 ± 9.4 years), acquired between January 2016 and November 2018 for assessment of cardiothoracic topography and structural changes were retrospectively analyzed. Depending on the physician’s recommendation, patients with a heart rate above 70 bpm were administered 10 or 40 mg propranolol or 40 mg verapamil before the procedure. The imaging parameters for CT were as follows: 100–120 kV tube voltage and 350–400 mA effective tube current. A contrast agent was injected at a dose of 1.0 mL/kg at a rate of 5 mL/s during imaging. The collimation was 2 × 32 × 0.6 mm and the temporal resolution was 165 ms. For the test bolus, the acquisition delay corresponded to the time of maximum density of the ascending aorta with an additional 6 s delay. The images were reconstructed with a B26f and B46f kernel and an image matrix of 512 × 512 pixels. The 70% phase of the multiphasic reconstruction (10% to 100%) was evaluated as the end-diastolic (ED) phase of the LV and further analyzed.

### 2.2. Image Processing and Analysis

**Figure 1:**
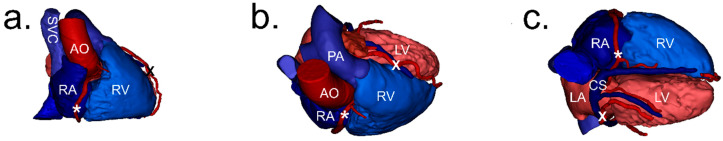
Visualization and 3D reconstructions of the tricuspid valve annulus region from (**a**) anteromedial, (**b**) superior, and (**c**) inferior views. AO—aorta, CS—coronary sinus, LA—right atrium, LV—left ventricle, PA—pulmonary trunk, RA—right atrium, RV—right ventricle, SVC—superior vena cava, X—left coronary artery. The right coronary artery is marked with *. This type of visualization can be advantageous in the preoperative planning of tricuspid valve interventions.

**Figure 2:**
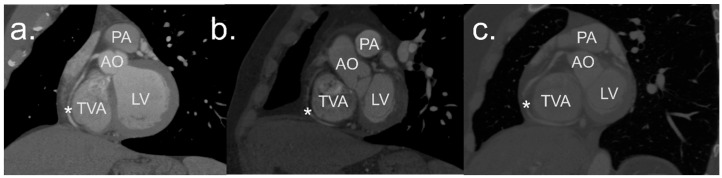
Multiplanar reconstruction of computed tomography angiography visualizing the tricuspid valve annulus area. (**a**) View of the tricuspid valve annulus region from RAO 60°, caudal 5°; (**b**) RAO 40°, caudal 0°; (**c**) RAO 45°, caudal 10°. The right coronary artery is marked with *. AO—aorta, PA—pulmonary trunk, TVA—tricuspid valve annulus, LV—left ventricle.

The RCA, its branches, ascending aorta, left atrium (LA), left ventricle (LV), right atrium (RA), and right ventricle (RV) were semiautomatically segmented, processed, and measured in the predefined ED phase using the visualization and three-dimensional reconstruction software Mimics Innovation Suite 24.0 (Materialize, Leuven, Belgium). The three-dimensional visualization of the TVA and the corresponding CTA scans used to create it are shown in Figure 1 and Figure 2.

### 2.3. Definitions 

**Figure 3:**
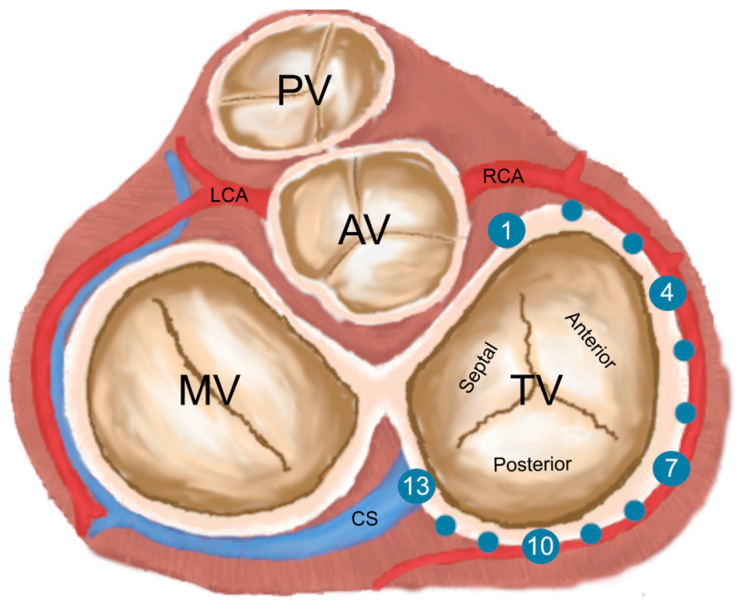
The distribution of the annular points T1–T13 (blue dots) is shown. In the projection of the commissure of the aortic root onto the TVA, T7 is located on the wall annulus between the incisions of the anterior and posterior valve leaflets, and T13 is located in the posterior region between the wall annulus and the septum. The vessels (RCA—right coronary artery, LCA—left coronary artery, CS—coronary sinus) and valves (TV—tricuspid valve, MV—mitral valve, AV—aortic valve, PV—pulmonary valve) are visible.

The TVA has been divided into two parts: a C-shaped wall ring formed by the free wall of the right ventricle (corresponding to the attachments of the anterior and posterior leaflets) and a septal ring, a shorter part corresponding to the attachment of the septal leaflet. Thirteen points (T1–T13) were placed on the circumference of the mural annulus: the first point (T1) was located anteriorly in the projection of the commissure of the aortic root onto the TVA; T7 was located on the mural annulus between the incisions of the anterior and posterior leaflets; T13 was placed posteriorly between the mural and septal annuli. Points T2–T12 were set at the same distances between T1–T7 and T7–T13 (Figure 3). The points corresponding to each tricuspid gyrus point were placed on the RCA. The TVA plane was defined as a plane intersecting the localized annular points (T1, T2, and T3). The RCA deviation was determined when the distance between the TVA plane and the RCA was greater than 10 mm. The atrial and ventricular deviations are RCA deviations closer to the right atrium or right ventricle. The “danger zone” was defined as a point with an RCA–TVA distance of less than 2 mm. This specific distance was selected based on the minimal space needed to place stitches and implant the annuloplasty ring. The lower distance increases the chance of occlusion caused by the TVA pressure on the artery and the probability of direct injury because of deep stitches placement [13,15,16] The “dangerous branch” was defined using the following criteria: the RCA branch with bifurcation point and TVA distance < 2 mm. 

### 2.4. Measurements

The circumference, the area, and the short (AP) and the long (ML) diameter of the TVA were measured. The shortest distance between the RCA and each tricuspid ring was measured. At predefined points of the tricuspid ring (T2, T4, T6, T8, T10, T12), it was checked whether the shortest distance between the RCA and the TVA plane was greater than 10 mm. The direction of RCA deviation was examined in six areas between selected ring points (T1–T3, T3–T5, T5–T7, T7–T9, T9–T11, T11–T13), distinguishing between atrial- and ventricular-directed deviation. The number of branches was examined in the six areas mentioned above.

### 2.5. Statistical Analysis

Data were analyzed using IBM SPSS Statistics 29.0 (Predictive Solutions, Pittsburgh, PA, USA). Categorical variables are presented as numbers (*n*) or percentages. Quantitative variables are presented as mean values with standard deviation. The normal distribution was analyzed using the Shapiro–Wilk test. Differences between normally distributed quantitative parameters were assessed using Student’s *t*-test and non-normally distributed quantitative data were tested using the Mann–Whitney *U*-test. Differences between categoric variables were assessed using the chi-square test for independence or the Fischer exact test if one of the groups had less than 5 observations. Correlations were tested with the Spearman’s rho correlation (two-tailed, α = 0.05, β = 0.2). A *p*-value < 0.05 was considered significant.

## 3. Results

### 3.1. TVA Dimensions

The measured mean ML diameter of the TVA was 43.1 ± 6.9 mm and the AP diameter was 41.8 ± 6.5 mm. The circumference of the TVA was 134.1 ± 14.8 mm and the area of the tricuspid valve was 1418.2 ± 317.2 mm^2^. The exact results are summarized in Table 1. 

### 3.2. TVA and RCA Distances

The shortest mean distances were measured at the following points: T9 (5.6 ± 3.1 mm), T10 (4.5 ± 2.8 mm), and T11 (4.9 ± 3.2 mm), and the longest at T1 (19.6 ± 5.5 mm). The exact distances at each point are listed in Table 1.

### 3.3. RCA Deviation

The ventricular-directed RCA deviation was present in 10.2% of the analyzed CTA scans. The atrial-directed RCA was observed in 5.9% of the cases. A detailed description of the RCA deviation at each tricuspid annular point can be found in Table 2.

### 3.4. Danger Zones

Hazardous zones were found in 20.56% of the cases studied. The highest prevalence of danger zones was found at tricuspid annular points located at the posterior leaflet insertions, at T10 (11.9% of cases) and T11 (9.2% of cases). The prevalence of danger zones was not sex- or age-dependent. A detailed description of the distribution of the danger zones can be found in Table 3.

### 3.5. RCA Branches

The number of RCA branches varied from 5 to 8. The number of branches in each part of the tricuspid annulus is described in Table 4. Dangerous branches were found in 3.7% of cases. The third coronary artery (conal branch origin in the aorta rather than in the RCA) was found in 16.51% of cases.

### 3.6. Gender-Specific Differences

In women, tricuspid valve leaflet parameters were smaller, including diameter, valve area, and annulus circumference. Women had a significantly greater distance between the TVA and RCA at T4. There were no gender differences in the prevalence of danger zones.

## 4. Discussion

### 4.1. The Dangerous Distances—Should We Be Aware of Them?

The reciprocal relationships between the TVA and RCA are crucial for intraoperative patient safety and for preventing iatrogenic complications, including fatal RCA occlusion. Our study provides dedicated, clinically oriented measurements of their topography. The smallest distance between the RCA and TVA and the most dangerous zones were observed in their posterior part, while the longest were found in the anterior region, near their ostium. There is no difference between men and women, which proves that both sexes are equally susceptible to iatrogenic complications related to surgery. In conclusion, the posterior part of the TVA is the main localization of its close mutual relationship with the RCA, so the part of the procedure performed in this region should be carried out with extreme caution.

### 4.2. Tricuspid Annular Points

In this article, we present the topographic course of the RCA in relation to the TVA. Analyzing the results will help avoid the fatal complications of RCA damage such as arrhythmias and hemodynamic instability in the peri- and postoperative period after tricuspid annuloplasty. Previous measurements of the TVA and RCA were mainly conducted in 2D planes and cadaver labs with smaller samples [2,13,17,18]. As far as the authors are concerned, there was only one proposition of the TVA topographic description based on measurements conducted by 3D segmented models up to this date. The mentioned study used magnetic resonance images as the base for segmentation and visualization [19]. The surgical topography approach (eight annular points) was chosen [19]. We propose a new TVA classification based on the localization of thirteen tricuspid annular points based on the TVA perimeter, allowing for an anatomical description of the TVA environment. Points T1–T6 correspond to the part of the mural annulus with the insertion of the anterior leaflet, T7 is the transition point between the anterior and posterior leaflets, and points T8–T13 are localized in the insertion zone of the posterior leaflet. This number of points was chosen to ensure the highest precision in describing the TVA and RCA relationships—both the anterior and posterior leaflets are divided into six parts by the localized points. This division is a compromise between the accuracy of the surgical description and the simplicity of the method. The distribution of the danger zones indicates that the central part of the posterior leaflet (T10, T11) is the most dangerous part of the mural ring where interventions can be performed, with the smallest measured distance between the TVA and RCA. 

### 4.3. Tricuspid Valve Diseases, Surgery and Its Complications

The defects of the tricuspid valve can be divided into two main types: regurgitation and extremely rare stenosis [20,21,22]. Stenosis (which occurs mainly in patients with rheumatic heart disease) accounts for only 1% of reported heart disease and can be treated with valvuloplasty [20]. TR is common in patients, especially in the elderly population [20]. Isolated TR is caused by various conditions, such as rheumatic heart disease, infective endocarditis, and congenital or iatrogenic conditions [20]. TR can also develop secondary to other cardiac abnormalities, such as pulmonary hypertension with RV remodeling, dilated cardiomyopathy, annular dilatation, or RV volume overload [20]. According to the 2020 ACC/AHA guideline, tricuspid valve annuloplasty should be performed primarily for left heart valve procedures in severe or progressive cases of TR or in progressive/severe TR with other specific conditions, including right heart failure, RV dilatation, or systolic dysfunction [20]. TR in mild stages is usually asymptomatic, and intervention during concomitant left-sided cardiac surgery may prevent the development of severe TR and heart failure [20]. The standard approach, sternotomy, may result in an unfavorable prognosis and high mortality compared with other isolated valve interventions [20]. Among other causes, the delay in surgical treatment and the decision made after the development of heart failure are among the most important factors affecting the poor survival rates in the treatment of TR [20]. The minimally invasive approach appears to have lower mortality and morbidity than the standard approach despite the lack of randomized controlled trials. The aforementioned close spatial relationship between the cardiac conduction system and the TVA is a major factor in the risk of iatrogenic complications during implantation of electronic cardiac devices: a cardioverter-defibrillator, cardiac resynchronization therapy defibrillator, pacemaker, or cardiac pacemaker [23]. Damage to the tricuspid valve can be caused by various mechanisms, such as pinching of the valve, restriction of valve movement, or perforation of the valve [23]. These iatrogenic injuries lead to valve regurgitation and further development of heart failure, resulting in increased patient mortality during post-procedure follow-up [23].

### 4.4. Surgical Topography of the Right Coronary Artery in the Region of the Tricuspid Valve Annulus

The TVA region is an anatomical area that is important for avoiding complications during tricuspid valve procedures, particularly because of its spatial relationship to the RCA. The RCA and its branches supply oxygenated blood to most of the RA and RV, the intraventricular septum, and parts of the LV [24]. Their occlusion leads to life-threatening acute myocardial infarction of the inferior wall of the LV with or without RV infarction. It is assumed that the damage to the RCA occurs mainly in the distal RCA segment after the right marginal branch [15]. Due to the small distances between the RCA and TVA in this region, the placement of deep stitches could cause direct arterial damage [15]. Another possible mechanism of occlusion could be the pressure of the TVA on the RCA caused by changes in annular shape. Regardless of the mechanism of injury, both could be increased in populations with TR, with shorter distances between the TVA and RCA [15]. This study showed that the typical RCA and TVA distance tended to decrease from T1 to T10 and increase from T10 to T13. The danger zones were located at T10 and T11. Presumably, the smallest distances at these points explain the highest occurrence of RCA damage in this region. The choice of more superficial stitches instead of deep stitches, especially in the danger zone, should be considered to prevent damage to the RCA. The results shown may suggest that a more detailed preoperative 3D assessment of the spatial relationships between the TVA and RCA increases the chance of recognizing the danger zone before planning the TVA procedure and, thus, preventing damage to the artery. While the effect of gender on heart size and tricuspid diameter was significant, there were no differences in the distances between the RCA and TVA. Similar anatomic distances between these structures suggest that representatives of both sexes are equally anatomically predisposed to RCA occlusion during tricuspid annuloplasty. To our knowledge, there has been only one cohort study addressing iatrogenic damage to the RCA, and a review of the literature found a total of only twelve cases (see Table 5), including six women, five men, and one case with unspecified sex of the patient [15,25,26,27,28]. Furthermore, it is impossible to say whether no anatomical differences correspond to no differences in complication prevalence. Age had no significant effect on the parameters of the TVA and most of the measured distances (*p* > 0.05). Only the RCA–TVA distance at T4 was related to age (*p* < 0.05), which is not clinically significant as no hazard zones were detected at this point. RCA deviation should be considered to prevent damage to an exceptionally localized artery, especially in the case of atrial RCA deviation due to the needle course when suturing from the atrial side and the invisibility of the site where the needle is pulled out. Cases of RCA injury were reported in DeVega annuloplasty (4), ring annuloplasty (4), and band annuloplasty (2). Two RCA occlusions were reported as a complication of a minimally invasive approach. It is uncertain whether this is because this method is one of the safest or because the fewest attempts have been performed. A previously mentioned review suggests that damage to the RCA during TAP is an underdiagnosed condition—only four cases were reported in the literature up to 2013 compared with four cases in seven years from one clinical center at the same time. Awareness of this complication is crucial. Recognition of RCA occlusion and emergency revascularization may be the only way to prevent patients’ death [15,25,26,27,28].

### 4.5. Clinical Implications

The distances between the RCA and TVA should be evaluated preoperatively using CTA imaging, especially in the distal part of the RCA (central and distal part of the posterior leaflet of the tricuspid valve) where most reported complications occurred and the highest prevalence of danger zones was found in our study. The more superficial stitches in danger zones should be considered. Extra care in planning and performing procedures in this part of the TVA could lead to a reduction in mortality and the number of complications. While the site of needle insertion is visible when the stitches are placed, the site of extraction is not visible to the surgeon. Therefore, the occurrence of deviations should be considered before suturing so as not to injure an exceptionally localized artery, especially in the case of atrial deviations.

### 4.6. Further Research Direction

To the extent that our study details the topography of the TVA region and the results indicate the most common locations of danger zones and the high need for their preoperative assessment, it lacks a strict correlation between the occurrence of RCA damage and other complications with specific variants of region anatomy. Retrospective evaluation of patients undergoing tricuspid valve annuloplasty, taking into account the specific anatomic features of each individual patient, could help establish more detailed protocols for the prevention of iatrogenic complications in tricuspid valve annuloplasty and presumably in other procedures in this region. The question of whether preoperative 3D visualization has a positive impact on tricuspid annuloplasty outcomes should also be investigated in further research to extend our thesis and develop a personalized, individualized approach for each patient. The CTA segmentation methods implemented in our study might be used to further investigate the clinical anatomy of different heart regions crucial for the prevention of iatrogenic complications [16,29,30].

## 5. Conclusions

Danger zones (distance < 2 mm between RCA and TVA) were found at the tricuspid annular points T9, T10, and T11, which may indicate that special care should be taken when planning interventions in this region. Presumably, a 3D model similar to the one created in this study could be helpful in preoperative, minimally invasive, and standard tricuspid annuloplasty planning as it accurately visualizes the course of the RCA, the deviation, and its spatial relationships to the TVA. Such visualization provides data that could be useful in decision making, especially in patients with morphological abnormalities, TVA dilatation, or non-normally distributed danger zones.

## Figures and Tables

**Table 1:** The tricuspid valve annulus and the right coronary artery in spatial relation. The points T1–T6 correspond to the invaginations of the anterior valve, T7 is the transition point between the anterior and posterior valve, and points T8–T13 correspond to the invaginations of the posterior valve. The table contains information on the TVA measurements and the distances between the TVA and RCA. Statistically significant differences are shown in bold.

Measurements, mm	Female	Male	General	*p*-Value
	Mean ± SD	Mean ± SD	Mean ± SD	
Age	64.8 ± 8.6	60 ± 9.5	62.1 ± 9.4	**0.002**
Short diameter	39.4 ± 6.4	43.8 ± 6	41.8 ± 6.5	**<0.001**
Long diameter	41.2 ± 5.9	44.6 ± 7.3	43.1 ± 6.9	**0.012**
Area	1279.2 ± 275.1	1527.3 ± 306.7	1418.2 ± 317.2	**<0.001**
Perimeter	127.2 ± 13.4	139.5 ± 13.7	134.1 ± 14.8	**<0.001**
T1	18.6 ± 5.2	20.5 ± 5.7	19.6 ± 5.5	**0.008**
T2	15.4 ± 4.6	15.6 ± 5.4	15.5 ± 5.1	0.889
T3	12.7 ± 4.3	11.2 ± 4.0	11.9 ± 4.2	0.073
T4	9.5 ± 4.0	8 ± 3.7	8.7 ± 3.9	**0.026**
T5	7.7 ± 3.1	7.1 ± 3.7	7.3 ± 3.4	0.197
T6	7.3 ± 2.7	6.9 ± 3.1	7.1 ± 2.9	0.384
T7	7.2 ± 3.3	6.3 ± 2.6	6.7 ± 2.9	0.229
T8	6.7 ± 3.0	6 ± 2.7	6.3 ± 2.9	0.379
T9	5.6 ± 2.8	5.5 ± 3.4	5.6 ± 3.1	0.648
T10	4.3 ± 1.6	4.7 ± 3.5	4.5 ± 2.8	0.783
T11	4.4 ± 1.9	5.3 ± 3.9	4.9 ± 3.2	0.462
T12	5.9 ± 2.7	6.4 ± 4.1	6.1 ± 3.6	0.683
T13	8.4 ± 3.7	9 ± 4.2	8.8 ± 4.0	0.509

**Table 2:** The right coronary artery deviations distribution and directions. RCA—right coronary artery.

RCA Deviation, Direction		Female	Male	General
		*n* (%)	*n* (%)	*n* (%)
T1–T3	none	46 (97.9)	57 (95)	103 (96.3)
	ventricular	1 (2.1)	3 (5)	4 (3.7)
T3–T5	none	46 (97.9)	59 (98.3)	105 (98.1)
	atrial	1 (2.1)	1 (1.7)	2 (1.9)
T5–T7	none	47 (100)	60 (100)	107 (100)
T7–T9	none	47 (100)	60 (100)	107 (100)
T9–T11	none	46 (97.9)	59 (98.3)	105 (98.1)
	atrial	1 (2.1)	1 (1.7)	2 (1.9)
T11–T13	none	41 (87.2)	56 (93.3)	97 (90.7)
	ventricular	4 (8.5)	3 (5)	7 (6.5)
	atrial	2 (4.3)	1 (1.7)	3 (2.8)

**Table 3:** The points with the highest prevalence of danger zones are printed in bold. Points T1–T6 correspond to the anterior leaflets, T7 is the transition point between the anterior and posterior leaflets, and points T8–T13 correspond to the posterior leaflets. No statistically significant differences were found.

Point	Male	Female	General	*p* Value
	*n* (%)	*n* (%)	*n* (%)	
T1	0 (0)	0 (0)	0 (0)	-
T2	0 (0)	0 (0)	0 (0)	-
T3	0 (0)	0 (0)	0 (0)	-
T4	0 (0)	1 (2)	1 (0.9)	0.114
T5	1 (1.6)	0 (0)	1 (0.9)	0.293
T6	0 (0)	0 (0)	0 (0)	0.168
T7	2 (3.2)	0 (0)	2 (1.8)	0.194
T8	4 (6.6)	0 (0)	4 (3.6)	0.686
**T9**	6 (9.8)	2 (4)	8 (7.2)	0.503
**T10**	8 (13.1)	5 (10)	13 (11.7)	0.1
**T11**	6 (9.8)	4 (8)	10 (9)	0.486
T12	3 (4.9)	3 (6)	6 (5.4)	0.863
T13	0 (0)	1 (2)	1 (0.9)	0.818

**Table 4:** Distribution of RCA branches based on tricuspid annular points.

Location	Number of Branches of RCA	*n* (%)
T1–T3	0	15 (13.9)
	1	52 (48.1)
	2	35 (32.4)
	3	6 (5.6)
T3–T5	0	6 (5.6)
	1	52 (48.1)
	2	38 (35.2)
	3	10 (9.3)
	4	2 (1.9)
T5–T7	0	7 (6.5)
	1	44 (40.7)
	2	44 (40.7)
	3	11 (10.2)
	4	2 (1.9)
T7–T9	0	39 (36.4)
	1	50 (46.7)
	2	16 (15)
	3	2 (1.9)
T9–T11	0	75 (70.1)
	1	32 (29.9)
T11–T13	0	2 (1.9)
	1	80 (74.8)
	2	24 (22.4)
	3	1 (0.9)

**Table 5:** Presentation of RCA damage cases reported in the literature.

Case	Age (y), Sex (M/F)	TVA Long Diameter (mm)	Surgery	Injured RCA Segment	Result
1	78, F	44	DeVega	Crux	Died
2	76, F	44	DeVega	Mid	Good
3	68, F	40	DeVega	Mid	Good
4	69, F	41	Ring	Distal	Good
5	N, N	N	Ring	N	Died
6	64, F	N	DeVega	Distal	Good
7	38, M	N	Band	Distal	Good
8	83, M	N	Band	Beyond RMA	Good
9	82, M	N	MI	Distal	Good
10	74, F	N	Band	Distal	Good
11	72, M	49	Ring	Distal	Good
12	44, M	N	MI	Distal	Good

N—unspecified, MI—minimally invasive annuloplasty, RMA—right marginal artery. The cases presented were found by reviewing the literature [15,16,17,18,19]. In patient number 1, revascularization was not possible during percutaneous coronary intervention, and the patient died as a result of cardiogenic shock. The mechanism of death and other information were not provided by the investigators reporting the case of patient number 5. The remaining patients survived after either a coronary bypass surgery or percutaneous coronary intervention.

## Data Availability

Data are available from the corresponding author upon reasonable request.
